# Supplementary material for: SORL1 Polymorphisms in Mexican Patients with Alzheimer’s Disease
Source: Genes (Basel). 2022 Mar 25;13(4):587. doi: 10.3390/genes13040587 (PMC9026506; doi:10.3390/genes13040587)
Supplement: Supplementary file 1 [file genes-13-00587-s001.zip › genes-1621620-supplementary.pdf]

Table S1. Single Nucleotide Polymorphisms (SNPs) used in this study. SNPs are referred in this paper according to Rogaeva *et al**.

Marker Number*	dbSNP rs Number	Alleles	Physical Map Location (bp)	SNP Type	Minor Allele (Frequency) White Subjects	African American Subjects
4	**rs661057**	C/T	120834164	Intrón 1	C (0.42)	C (0.29)
8	**rs668387**	T/C	120873131	Intrón 6	T (0.41)	T (0.36)
9	**rs689021**	G/A	120876330	Intrón 6	A (0.42)	A (0.35)
10	**rs641120**	C/T	120886175	Intrón	T (0.41)	T (0.33)
12	**rs12285364**	C/T	120898436	Intrón 9	T (0.05)	T (0.09)
19	**rs2070045**	G/T	120953300	Exón 25	G (0.23)	G (0.08)
20	**rs3824966**	C/G	120953393	Intrón	G (0.23)	G (0.08
22	**rs1699102**	C/T	120962172	N1246N	C (0.33)	T (0.47)
23	**rs3824968**	A/T	120981132	A1584A	A (0.31)	A (0.13)
24	**rs2282649**	C/T	120984168	Intrón 38	T (0.28)	T (0.11)
25	**rs1010159**	A/G	120988611	Intrón 39	C (0.37)	T (0.46)
26	**rs1784933**	A/G	120994626	Intrón	G (0.06)	G (0.39)

Figure S1

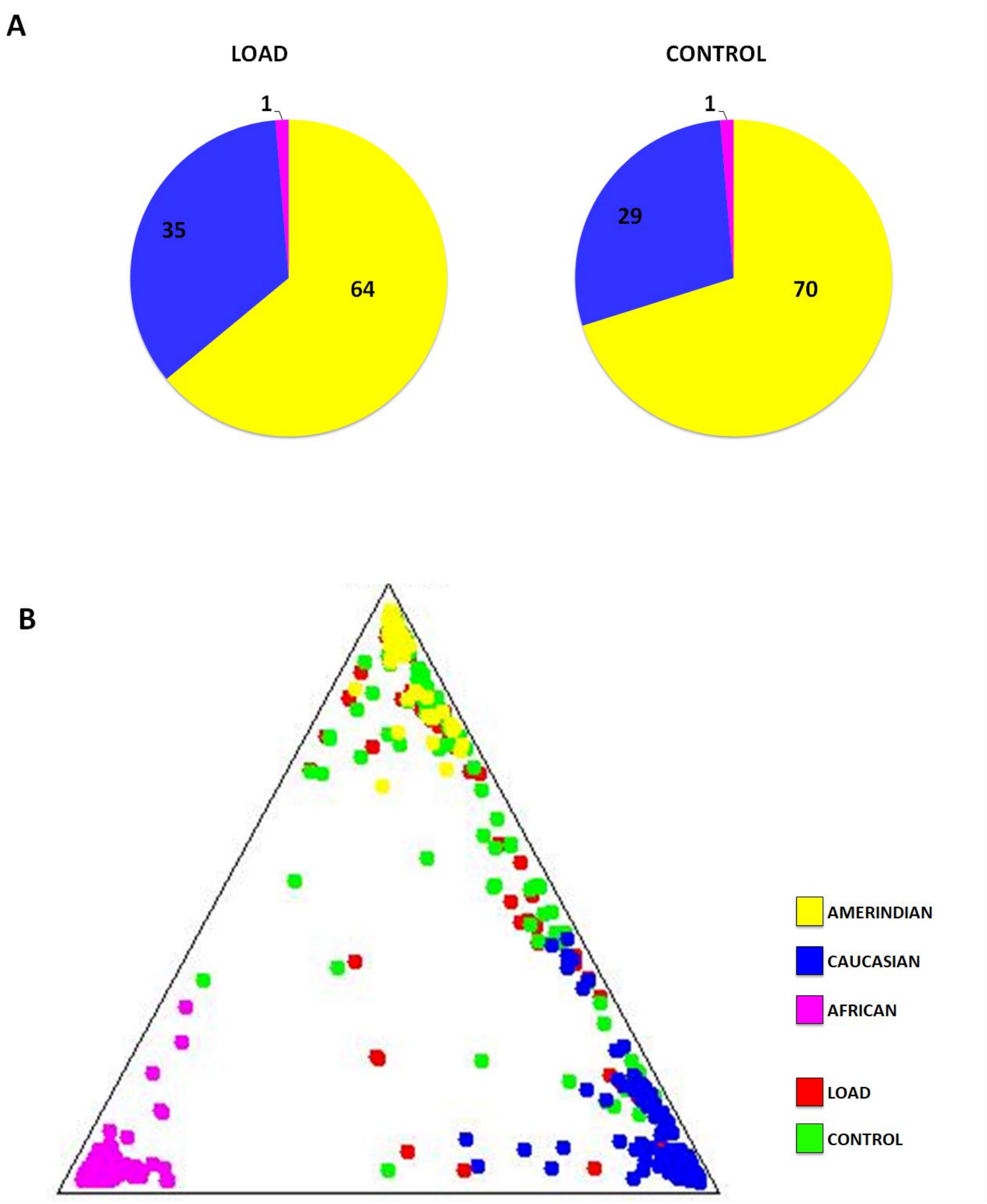

Figure S1. Cluster analysis and ancestry.
(A) The proportions of ancestry in each group of study were estimated in the LEADMIX software (Wang, 2003) taking the ancestral populations. No significant differences were found, the chi-square statistic is 0.8312. The *p*-value is 0.66. The result is *not* significant at $p < 0.05$. **(B)** The triangle obtained from the STRUCTURE software (Pritchardetal.,2000) allows to observe that the distribution of the individuals of each study group between the three ancestral clusters is similar.

Table S2. Genotypic distribution in different populations

		*CAUCASIAN [13,41]		**ASIAN [34,52-54]		***AFRICAN DESCENT [41]		****HISPANIC [41]		MEXICAN	
	GENOTYPE	CONTROL	LOAD	CONTROL	LOAD	CONTROL	LOAD	CONTROL	LOAD	CONTROL	LOAD
rs668387	CC	0.28	0.34	0.257	0.218	0.468	0.398	0.366	0.382	0.308	0.301
	CT	0.55	0.47	0.461	0.503	0.43	0.466	0.479	0.472	0.516	0.545
	TT	0.17	0.18	0.282	0.278	0.101	0.138	0.155	0.146	0.176	0.154
rs689021	GG	0.28	0.34	0.302	0.304	0.456	0.375	0.325	0.365	0.299	0.308
	GA	0.55	0.47	0.461	0.492	0.437	0.477	0.49	0.478	0.525	0.545
	AA	0.17	0.19	0.237	0.204	0.108	0.148	0.186	0.157	0.176	0.147
rs641120	GG	0.28	0.34	0.302	0.272	0.551	0.472	0.387	0.421	0.308	0.314
	GA	0.55	0.46	0.574	0.528	0.38	0.427	0.469	0.455	0.52	0.538
	AA	0.17	0.19	0.12	0.20	0.07	0.101	0.144	0.124	0.172	0.147
rs2070045	GG	0.05	0.047	0.26	0.182	0.019	0.011	0.057	0.056	0.308	0.269
	GT	0.391	0.375	0.60	0.519	0.228	0.18	0.361	0.354	0.466	0.487
	**TT**	0.559	0.578	**0.14**	**0.299**	0.753	0.809	0.582	0.59	**0.226**	**0.244**
rs3824966	GG	0 (-)	0 (-)	0.613	0.565	0 (-)	0 (-)	0 (-)	0 (-)	0.294	0.269
	GC	0 (-)	0 (-)	0.336	0.363	0 (-)	0 (-)	0 (-)	0 (-)	0.462	0.474
	CC	0 (-)	0 (-)	**0.051**	**0.072**	0 (-)	0 (-)	0 (-)	0 (-)	**0.244**	**0.256**
rs1699102	CC	0.113	0.102	0.615	0.561	0.234	0.216	0.227	0.219	0.466	0.397
	CT	0.481	0.463	0.346	0.356	0.50	0.5	0.5	0.5	0.416	0.436
	TT	0.406	0.435	0.038	0.083	0.266	0.284	0.273	0.281	0.118	0.167
rs3824968	TT	0.065	0.097	0.15	0.08	0.025	0.023	0.093	0.079	0.213	0.244
	TA	0.387	0.437	0.47	0.44	0.259	0.239	0.423	0.404	0.493	0.493
	AA	0.548	0.466	0.38	0.49	0.715	0.739	0.485	0.517	0.294	0.263
rs2282649	TT	0.043	0.088	0.195	0.229	0.025	0.011	0.077	0.079	0.294	0.25
	TC	0.333	0.422	0.501	0.558	0.278	0.193	0.402	0.404	0.471	0.487
	CC	0.624	0.49	0.304	0.213	0.696	0.795	0.521	0.517	0.235	0.263
rs1010159	CC	0.14	0.117	0.202	O.227	0.31	0.352	0.211	0.23	0.321	0.276
	CT	0.473	0.447	0.496	O.558	0.494	0.477	0.495	0.5	0.452	0.314
	TT	0.387	0.437	0.302	0.215	0.196	0.17	0.294	0.27	0.226	0.41
rs1784933	GG	0.013	0.034	0.64	0.461	0.019	0.11	0.026	0.034	0.226	0.154
	GA	0.158	0.292	0.32	0.423	0.228	0.18	0.273	0.292	0.452	0.41
	**AA**	0.829	0.874	**0.04**	**0.116**	0.753	0.809	0.701	0.674	**0.321**	**0.436**

*Mixed populations

Table S3. Allelic Distributions in different populations

		*CAUCASIAN [13,41]		**ASIAN [34,52-54]		***AFRICAN DESCENT [41]		****HISPANIC [41]		MEXICAN	
	ALLELE	CONTROL	LOAD	CONTROL	LOAD	CONTROL	LOAD	CONTROL	LOAD	CONTROL	LOAD
**rs668387**	**C**	0.55	0.58	0.49	0.47	0.68	0.63	0.61	0.62	0.57	0.57
	**T**	0.45	0.42	0.51	0.53	0.32	0.37	0.39	0.38	0.43	0.43
**rs689021**	**G**	0.55	0.57	0.53	0.55	0.67	0.61	0.57	0.6	0.56	0.58
	**A**	0.45	0.432	0.47	0.45	0.33	0.39	0.43	0.4	0.44	0.42
**rs641120**	**G**	0.55	0.58	0.59	0.54	0.74	0.69	0.62	0.65	0.57	0.58
	**A**	0.45	0.42	0.41	0.46	0.26	0.31	0.38	0.35	0.43	0.42
**rs2070045**	**G**	0.24	0.23	0.56	0.44	0.13	0.1	0.24	0.23	0.54	0.51
	**T**	0.76	0.77	**0.44**	**0.56**	0.87	0.9	0.76	0.77	**0.46**	**0.49**
**rs3824966**	**G**	0 (-)	0 (-)	0.781	0.747	0 (-)	0 (-)	0 (-)	0 (-)	**0.47**	**0.49**
	**C**	0 (-)	0 (-)	0.219	0.253	0 (-)	0 (-)	0 (-)	0 (-)	0.53	0.51
**rs1699102**	**C**	0.33	0.35	0.79	0.74	0.48	0.47	0.48	0.47	0.67	0.61
	**T**	0.67	0.65	0.21	0.26	0.52	0.53	0.52	0.53	0.33	0.39
**rs3824968**	**T**	0.26	0.4	0.41	0.31	0.16	0.14	0.3	0.28	0.46	0.49
	**A**	0.74	0.6	0.59	0.69	0.84	0.86	0.7	0.72	0.54	0.51
**rs2282649**	**T**	0.2	0.32	0.45	0.51	0.16	0.11	0.28	0.28	0.53	0.49
	**C**	0.8	0.68	0.55	0.49	0.84	0.89	0.72	0.72	0.47	0.51
**rs1010159**	**C**	0.38	0.42	0.45	0.51	0.56	0.59	0.46	0.48	0.55	0.48
	**T**	0.62	0.58	0.55	0.49	0.44	0.41	0.54	0.52	0.45	0.52
**rs1784933**	**G**	0.09	0.07	0.8	0.672	0.37	0.26	0.16	0.18	0.45	0.36
	**A**	0.91	0.93	**0.2**	**0.328**	0.63	0.74	0.84	0.82	**0.55**	**0.64**

***Mixed populations**

Table S4. Haplotype analysis

**Haplotype association with response (n=377, adjusted by AGE+GENDER+ANCESTRY)**												
**rs668387**	**rs689021**	**rs641120**	**rs2070045**	**rs3824966**	**rs1699102**	**rs3824968**	**rs2282649**	**rs1010159**	**rs1784933**	**Freq**	**OR (95% CI)**	**P-value**
C	G	C	G	G	C	A	T	G	G	0.1528	1	---
T	A	T	G	G	C	A	T	G	A	0.1388	0.78 (0.39 - 1.59)	0.5
C	G	C	T	C	T	T	C	A	A	0.1068	1.26 (0.62 - 2.56)	0.52
**T**	**A**	**T**	**T**	**C**	**T**	**T**	**C**	**A**	**A**	**0.0967**	**0.37 (0.17 - 0.81)**	**0.014**
C	G	C	G	G	C	A	T	G	A	0.0737	1.00 (0.47 - 2.13)	0.99
C	G	C	T	C	T	T	C	A	G	0.0716	0.88 (0.37 - 2.09)	0.77
**T**	**A**	**T**	**G**	**G**	**C**	**A**	**T**	**G**	**G**	**0.059**	**4.92 (1.40-17.28)**	**0.013**
C	G	C	T	C	C	T	C	A	G	0.0321	0.87 (0.26 - 2.89)	0.82
C	G	C	T	C	C	T	C	A	A	0.0296	0.80 (0.24 - 2.62)	0.71
T	A	T	T	C	C	T	C	A	A	0.023	4.61 (0.54 - 39.28)	0.16
T	A	T	G	G	C	A	T	A	A	0.0168	0.75 (0.24 - 2.29)	0.61
T	A	T	T	C	T	T	C	G	A	0.0125	1.23 (0.22 - 6.91)	0.82
*	*	*	*	*	*	*	*	*	*	0.1865	1.02 (0.57 - 1.85)	0.94
**Global haplotype association p-value:** 0.053												

Table S5. Three-loci haplotype frequency in AD and controls. No association with the presence of (8-9-10), (19-22-23) and (22-23-24) haplotypes in Mexican patients.

Marker No.a	HAPLOTYPE	HAPLOTYPE FREQUENCY		OR (95% CI)	*Haplotype P-value*
		AD	Controls		
**8-9-10**	**C G C**	0.5574	0.5496	1	-
	**T A T**	0.4004	0.4184	1.06 (0.77-1.46)	0.71
	**T A C**	0.0097	0.0137	1.33 (0.37-4.77)	0.66
	**C G T**	0.0065	0.0092	-	-
	**T G C**	0.0162	0.0023	-	-
	**C A T**	0.0098	0.0045	-	-
	**C A C**	NA	0.023	-	-
**19-22-23**	**G C A**	0.4723	0.4982	1	-
	**T T T**	0.363	0.3104	0.82 (0.59-1.12	0.22
	**T C T**	0.0943	0.1063	1.12 (0.66-1.91)	0.67
	**T C A**	0.0224	0.0398	1.73 (0.67-4.46)	0.26
	**G C T**	0.0263	0.0299	1.10 (0.42-2.85)	0.85
	**G T T**	0.0068	0.0126	1.60 (0.35-7.34)	0.55
**22-23-24**	**C A T**	0.4763	0.5178	1	-
	**T T C**	0.37	0.3184	0.81 (0.59-1.12)	0.21
	**C T C**	0.1105	0.1293	1.13 (0.70-1.85)	0.61
	**C A C**	0.0187	0.0201	1.36 (0.44-4.21)	0.59
	**C T T**	0.0099	0.077	-	-
	**T A C**	0.0072	0.0028	-	-
